# Magnetic Resonance Cholangiopancreatography with Secretin Stimulation in the Diagnosis of Intraductal Papillary Mucinous Neoplasm: A Paradigmatic Case Report

**DOI:** 10.1155/2014/820359

**Published:** 2014-02-13

**Authors:** Elsa Iannicelli, Francesco Carbonetti, Marco Di Pietropaolo, Giulia Francesca Federici, Gabriele Capurso, Vincenzo David

**Affiliations:** ^1^Sant'Andrea Hospital, Rome, Department of Radiology, Faculty of Medicine and Psychology, Sapienza University of Rome, Via Di Grottarossa, 1035-00135 Rome, Italy; ^2^Sant'Andrea Hospital, Rome, Department of Gastroenterology, Faculty of Medicine and Psychology, Sapienza University of Rome, Via Di Grottarossa, 1035-00135 Rome, Italy

## Abstract

*Context.* One of the characteristic findings of intraductal papillary mucinous neoplasms (IPMN) is the presence of a direct communication between the lesion and the ductal pancreatic system and when magnetic resonance cholangiopancreatography (MRCP) shows uncertain findings, it is useful to perform a MRCP after secretin stimulation (MRCP-S) which provides a better visualization of the ductal system. *Case Report.* We present a case of 51-year-old man in whom, during a CT follow-up for a renal tumour, was found a cystic lesion of the pancreas. To better evaluate the lesion and its suspected communication with the pancreatic system, MR with gadolinium and MRCP and MRCP-S were performed. With the MRCP and MRI it was not possible to identify a clear communication between the cystic lesion and the ductal system. MRCP-S showed an increase in signal intensity of the lesion and its communication with the ductal system, allowing us to classify the cystic lesion as a main duct in intraductal papillary mucinous neoplasm. The patient underwent a surgical duodenal pancreatectomy. The histological result of the specimen confirmed the diagnosis of adenocarcinoma IPMN. *Conclusion.* In this case MRCP-S has allowed a clearer identification of the cystic lesion allowing a correct diagnosis and treatment.

## 1. Introduction

During the past year an increase of cystic pancreatic lesions, due to the widespread use of cross-sectional imaging, was observed. A correct assessment of pancreatic cystic lesions plays an important role as the different degree of malignancy of the lesions and therefore their different medical treatment.

Imaging plays a fundamental role in the correct diagnosis and classification of pancreatic cystic lesions.

A good evaluation of the morphology of the ductal system permits visualizing the signs of a chronic pancreatitis and its related pseudocysts, which are the most common cause of pancreatic cystic lesion.

In the evaluation of the pancreatic cystic neoplasms (mucinous cystadenoma, serous cystadenoma, and IPMN with different degree of atypia) it is important to have a clear view of the relationship that the lesion has with the ductal system, since the presence of a communication between the lesion and the ductal system is a typical finding of intraductal papillary mucinous neoplasms (IPMNs). The gold standard for the study of the ductal system is magnetic resonance cholangiopancreatography (MRCP), yet sometimes it can not provide unique information on the relationship of the cystic lesions with the ductal system. MRCP with secretin stimulation provides a clearer view of the ductal system and of its relations with cystic lesion of the pancreas, thus allowing better diagnostic classification of the IPMN.

## 2. Case Report

**Figure 1:**
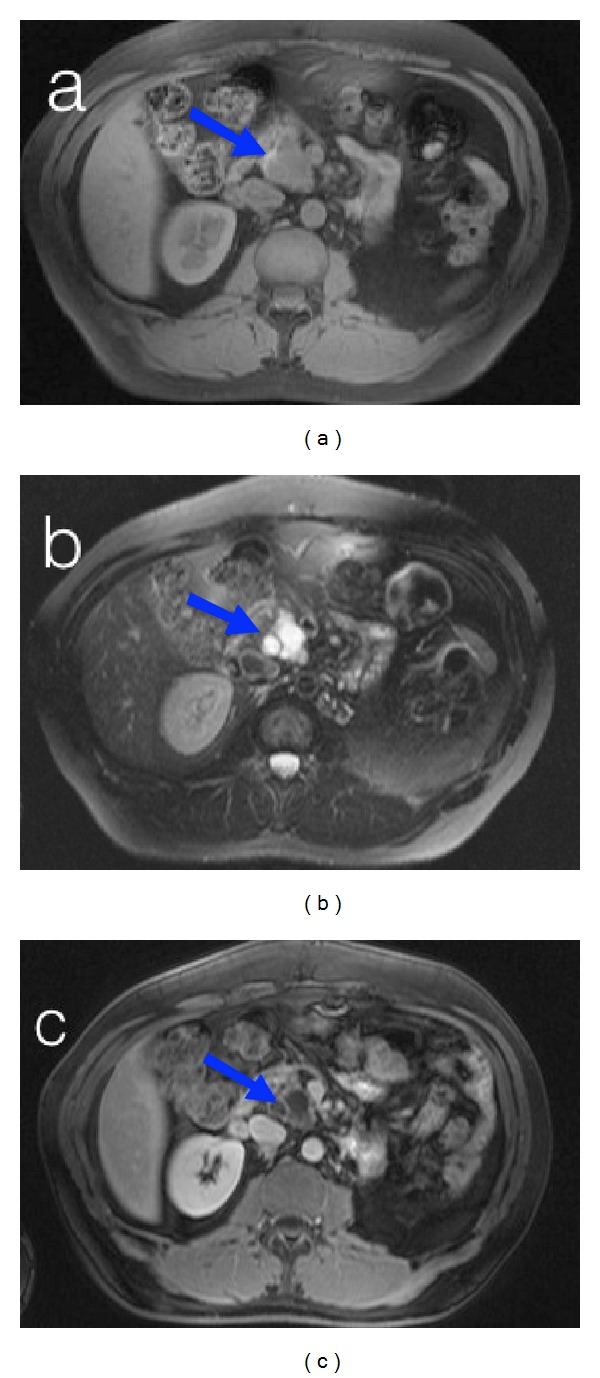
The scans show a multichambered lesion, 3.3 × 2.5 cm in size, localized in the head and in the uncinate process of the pancreas, hypointense in T1-weighted image (a) and hyperintense in T2-weighted image (b). After the intravenous contrast medium administration (c), the enhancement of the capsule and internal septa was shown. (a) Precontrast axial T1-WI FAT SAT. (b) Precontrast axial T2-WI FAT SAT. (c) Postcontrast axial T1 WI. The blue arrow indicates the lesion.

A 51-year-old man with a previous history of renal neoplasm was admitted to our department in order to perform a computed tomography (CT) scan for follow-up. The CT scan showed the presence of a pancreatic lesion, localized in the head and in the uncinate process of the pancreas. The patient was completely asymptomatic and did not report any previous symptoms that could be related with the pancreatic lesion. In order to characterize the lesion, an upper abdomen magnetic resonance (MR) before and after the administration of contrast medium was performed. MR examination was carried out with 1.5 Tesla equipment with a four-single phased array coil positioned on the patient's upper abdomen. The MRI protocol involved the use of T1-wheighted gradient echo (GRE) sequences with in-phase and out-of-phase echo-time, T1-weighted fat-suppressed GRE sequences, and T2-weighted half-Fourier single-shot turbo spin-echo (HASTE-TSE). The dynamic study was performed during administration of 0.1 mmol/kg body weight of gadolinium chelates with a four-phase technique: precontrast, pancreatic phase (30–40 s), portal venous phase (80 s), and delayed phase (180 s). The dynamic study used a T1-weighted 3D GRE volumetric interpolated breath-hold examination (VIBE) sequence with chemically selective fat saturation in the axial plane. The MRI revealed a multilobulated lesion, hyperintense in T2 WI and hypointense in T1 WI, 3.3 × 2.5 cm in size, and it is localized in the head and in the uncinate process of the pancreas (Figure 1). After the administration of contrast medium this lesion showed an enhancing capsule and internal septae. Moreover the Wirsung duct was 9 mm in size, presenting an irregular morphology with dilatation of the side branches.

**Figure 2:**
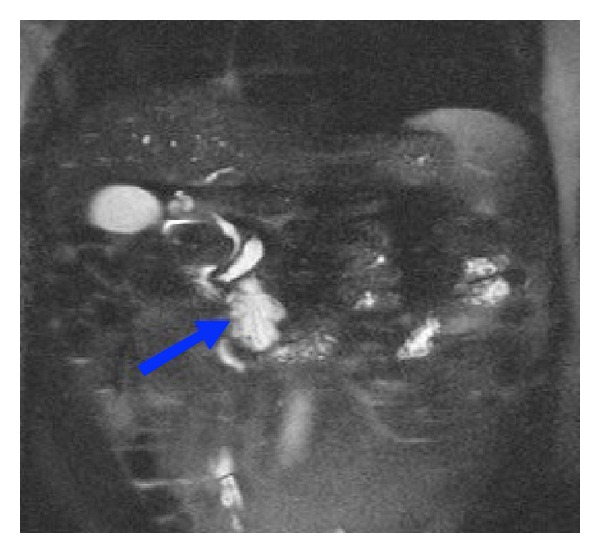
MRCP Coronal HASTE 2D. The scan shows the fusiform dilatation of the Wirsung duct at the head of the pancreas and the cystic lesion. The blue arrow indicates the lesion.

**Figure 3:**
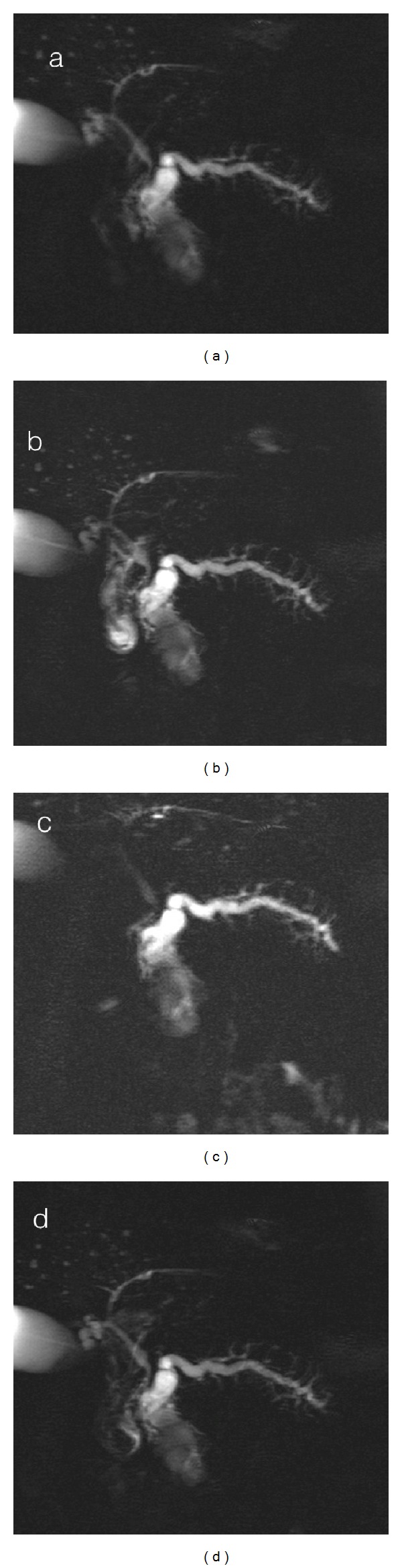
Single shot T2 WI before (a) and after ((b)-(c)-(d)) secretin stimulation show an increase of the signal intensity of the Wirsung duct of the cystic lesion; the communication of the lesion with the Wirsung duct is now clearly visible and is also possible to appreciate the signs of acinar depletion of the branch ducts.

To better assess the findings charged to the Wirsung the patient underwent MRCP (Figure 2) and MRCP with secretin stimulation (MRCP-S) (Figure 3). The MRCP was performed using a 2D single-slab rapid acquisition in coronal planes and 3D HASTE sequence with respiratory triggering in a coronal oblique plane depending on the course of the main pancreatic duct as visualized on a 2D sequence acquired in the axial plane. The 3D HASTE source images are analysed and subsequently processed with thin MIP algorithm and multiplanar reconstruction algorithm (MPR). Following basal MRCP evaluation, secretin (Screlux; Goldhman-Bioglan, Zusmarhausen, Germany) was administered intravenously at the dose of 1 mL/Kg of body weight, after the administration per os of a superparamagnetic contrast agent in order to put down the intestinal fluids' signal and T2-steady-state sequences are repeated every 30 seconds for 10 minutes.

MR scans clearly showed the communication of the lesion with the main pancreatic duct (MPD); in addition after secretin stimulation the cystic lesion showed an increase in signal intensity, confirming the lesion's origin from the pancreatic duct system. Moreover the MR showed irregular and multiple dilatations of the branch ducts and reduction of thickness of the pancreatic parenchyma.

**Figure 4:**
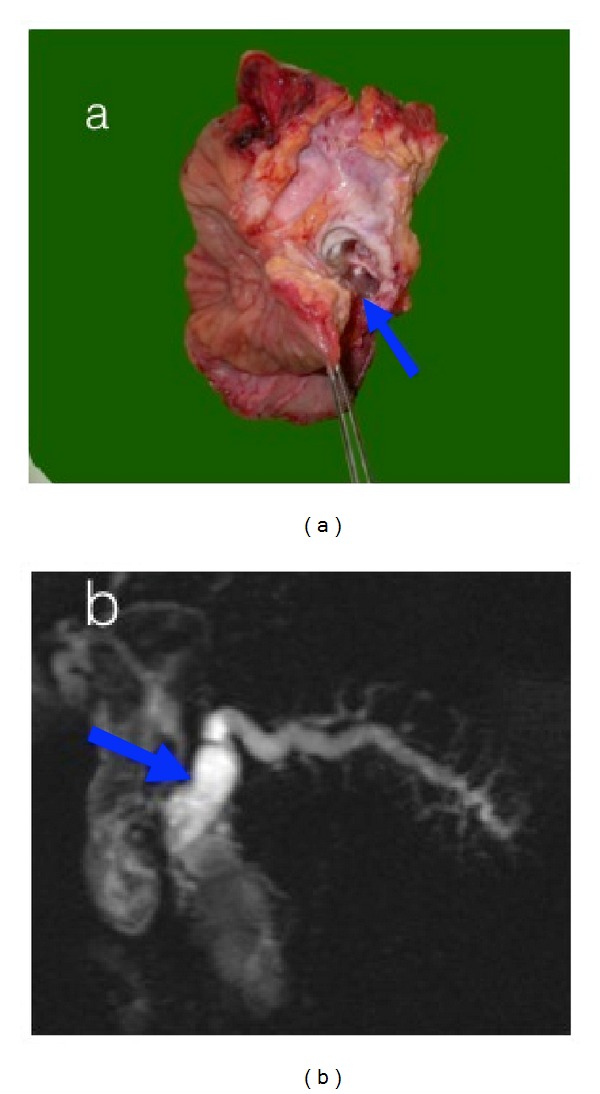
Surgical specimen is possible to appreciate a clear correspondence between the specimen (a) and the imaging findings (b). The histological result was IPMN adenocarcinoma. The blue arrow indicates the lesion.

Therefore, MRCP-S allowed us to classify the cystic lesion as a main duct IPMN. Due to the high risk of malignant transformation of the main duct IPMN, the patient underwent a surgical duodenal pancreatectomy to remove the lesion and a hepatic-jejunal anastomosis was made. The histological result of the specimen (Figure 4) confirmed the diagnosis of adenocarcinoma IPMN.

## 3. Discussion

IPMNs are cystic neoplasms arising from the epithelial lining of the pancreatic ductal system; they represent the 10% of pancreatic cystic lesions and 5% of all cancers of the exocrine pancreas. They are characterized by papillary growth, overproduction of mucin, and direct communication with the ductal system. The overproduction of mucin expands the duct and causes the cystic lesion, usually associated with parenchymal atrophy. The neoplastic cells show a variety of atypia ranging from low to high grade, which leads to a diagnosis of adenoma or adenocarcinoma depending on the atypical degree [1].

Usually patients are asymptomatic, and the IPMN is found accidentally, or, less frequently, patients present nonspecific symptoms such as acute pancreatitis, nausea, vomiting, diabetes, or jaundice. Chronic pancreatitis is often associated with IPMNs [2].

IPMNs are subclassified as main and branch duct types and as a mixed type that contains elements of both. Main duct IPMN is characterized by involvement of the duct of Wirsung, which is dilated to more than 1 cm in diameter. Branch duct IPMN originates in the side branches of the pancreatic ductal system and appears as cystic lesion communicating with a non dilated main pancreatic duct. If the main duct is dilated with synchronous involvement of the branch ducts, it is described as mixed IPMN.

The risk of malignant degeneration is different according to the type of tumour; IPMN main duct has a risk of 70%, IPMN branch duct has a risk of 25%, and IPMN mixed type has the same risk of the main duct [3].

Their different biological behaviour translates into different treatment approaches, with main duct IPMN and mixed type IPMN being candidates for surgical treatment and branch duct IPMN for close monitoring over time [4].

Ideally, the imaging modality to evaluate IPMNs at baseline and follow-up should provide adequate information regarding the size of the lesion, the size of the main pancreatic duct, the presence of communication between the lesion and the ductal system, and the presence of intramural node. Patients with a suspect of IPMN, found during other examinations (i.e., US or CT), must undergo abdominal MRI and MRCP to better assess the nature of the lesion.

When MRCP fails to identify a clear communication between the lesion and the pancreatic duct, MRCP-S should be performed to better evaluate the relationship of the lesion with the ductal system.

The exogenous administration of secretin stimulates fluid and bicarbonate secretion by the exocrine pancreas; consequently, the volume of fluid in the pancreatic ductal system and in the duodenum increases. Because these changes increase the T2 signal intensity of the pancreatic duct, MRCP in combination with the administration of secretin may allow better evaluation of the pancreatic ductal system, facilitating the visualization of the lesion's communication with the ductal system, and allows better evaluation of the signs of chronic pancreatitis. Furthermore, with the secretin stimulation it is also possible to evaluate the increase of the signal intensity of the lesion, which confirms its origin from the ductal system. Thus the transient increase in the diameter of the pancreatic duct after the secretin stimulation improves the depiction of the ductal anatomy, which can be useful in patients in whom detailed evaluation of the pancreatic duct is most desired because of a suspicion of pancreatic disease. Improved depiction of the ductal anatomy can be important [5].

MRCP with secretin stimulation visualizes also the amount of pancreatic fluid secreted into the duodenum and so allows an indirect and semiquantitative estimation of the pancreatic exocrine function, which is useful information for the clinical management of the chronic pancreatic diseases [6].

In our case the cystic lesion was found with the CT scan and was confirmed by MR imaging; MRCP clearly depicted the anatomy of the Wirsung and the shape of the lesion; after the secretin stimulation the presence of communication between the lesion and the main duct was found, and it was also possible to detect the signs of chronic pancreatitis charged to the branch ducts and to evaluate the pancreatic function, and an increase of the signal intensity of the cystic lesion was found confirming the origin of the cystic lesion from the ductal system. In our case the additional findings obtained with the MRCP with secretin stimulation permitted us to diagnose a main duct IPMN, which was not possible to diagnose just with the MRCP. The patient thanks to the findings obtained with CPRM-S and due to the high risk of malignant transformation of main duct IPMN immediately underwent surgery. In the case reported MRCP-S permitted us to obtain further information fundamental for a correct diagnosis and for a prompt surgical intervention. Due to MRCP-S higher capacity to evaluate the pancreatic ductal system and due to the different biological behavior of IPMNs, CPRM-S is a useful exam in the management of IPMNs and it must be performed in all suspected cases of IPMNs where the MRCP fails to identify a clear communication of the lesion with the pancreatic duct.

## Abbreviations

CE-MR: Contrast enhanced magnetic resonance
FAT-SAT: FAT saturation
GRE: Gradient echo
HASTE-TSE: Half-Fourier single-shot turbo spin-echo
MIP: Maximum intensity projection
MPR: Multiplanar reconstruction algorithm
MRCP: Magnetic resonance cholangiopancreatography with secretin stimulation
MRCP-S: Magnetic resonance cholangiopancreatography with secretin stimulation
IPMN: Intraductal papillary mucinous tumors of the pancreas
T2 WI: T2-weighted image
T1 WI: T1-weighted image
VIBE: Volumetric interpolated breath-hold examination.
